# The preliminary experience of methylene blue assisted laparoscopy in the treatment of renal parapelvic cysts

**DOI:** 10.1038/s41598-020-76006-4

**Published:** 2020-10-30

**Authors:** Zhen Ma, Song Li, Fang-Min Chen, Da-Hai Yu, Xiao-Guang Zhang, Kai Li, Ming-Hao Zhang, Shuai Tang, Qi Wang

**Affiliations:** Department of Urology, Tianjin Third Central Hospital, NO.83 Jintang Road, Hedong District, Tianjin, 300170 China

**Keywords:** Diseases, Medical research, Urology

## Abstract

Renal cyst is a common disease in humans and laparoscopic renal cyst decortication is the gold standard for treatment. However, specialized surgical skills are required for the treatment of renal parapelvic cysts. In this study, we describe an improved laparoscopic method for the treatment of renal parapelvic cysts involving the use of continuous infusion of methylene blue. Sixteen patients with renal parapelvic cyst were enrolled in this study. All patients underwent retrograde ureteral catheterization, with continuous perfusion of the renal pelvis using a solution of 0.2% methylene blue and saline, during laparoscopic decortication of the parapelvic cyst. In one patient, the cyst communicated with the renal collection system which was injured, but this was immediately repaired intraoperatively. All operations were successful, and none was converted to open surgery. There were no occurrences of persistent urinary fistula, bleeding, or other complications postoperatively. All patients were followed-up for 3–24 months, and results of postoperative imaging investigations revealed that all of our patients experienced either complete recovery or a greater than 50% decrease in size of the cysts. Our study demonstrates that methylene blue-assisted laparoscopic treatment is a safe, effective and practical method for the treatment of renal parapelvic cysts.

## Introduction

Renal cysts are the most common lesion of the kidney^1^, with a prevalence of 20–50% in the general population^2^. The incidence of renal cysts increases with age^3^. Most are simple renal cysts and are often asymptomatic. By comparison, renal parapelvic cysts are less common, with an incidence of approximately 1–3%^4^; however, they are more likely to be symptomatic^5^. Laparoscopic decortication of renal cysts is one of the most commonly used surgical methods. However, renal parapelvic cysts are usually located close to the collecting system, which makes them difficult to find and prone to injury during surgery. Therefore, some serious complications may occur after surgery, such as infection, long-term urinary leakage, and even nephrectomy. However, studies on how to identify the relationship between cysts and the collecting system during surgery in order to reduce the incidence of damage to the renal collecting system, are currently rare. In this study, we used methylene blue to aid in distinguishing the cysts from the renal collection system, in order to find and repair the damaged collection system quickly during surgery, to avoid postoperative urinary fistula or secondary surgery, and to achieve other results, as described below.

## Methods

### Study population

This study was approved by the Ethical Committee of the Third Central Hospital of Tianjin, and all methods were performed in accordance with the relevant guidelines and regulations. All patients provided written informed consent before participating in this study. Between May 2017 and December 2019, a total of 115 cases of renal cysts were treated, comprising 16 cases of renal parapelvic cysts (13.9%) that were included in this study. The inclusion criteria were: (1) symptoms, such as lumbago, hematuria, renal stones, and a history of repeated infection; and (2) renal cyst larger than 4 cm in diameter, or cysts that increases rapidly in volume over a short duration”. The exclusion criteria were: (1) Bosniak III–V complex renal cyst; (2) completely endogenous renal parapelvic cyst; and (3) patients with poor physical condition that were not fit for surgery. The basic information of the included cases are shown in Table 1. The patients included 9 males and 7 females; the mean age was 59.9 years (range, 41–74 years).The mean maximum diameter of the cysts was 6.6 cm (range, 4.8–10.5 cm). Eight patients presented with flank pain, and the other eight were asymptomatic patients that were incidentally identified on physical examination. One patient was diagnosed with an ipsilateral renal cell carcinoma, with no association between the renal cell carcinoma and the location of the cyst. One patient had ipsilateral kidney stones (0.6 cm), six patients had ipsilateral hydronephrosis, four patients also had ipsilateral simple renal cysts. All patients were diagnosed using color doppler abdominal ultrasonography, enhanced computed tomography urography and/or intravenous urography prior to surgery. Laparoscopic renal cyst decortication was performed with the assistance of continuous methylene blue ureteral infusion.

**Table 1:** Patients’ characteristics.

Number	Sex	Age (years)	Preoperative Cyst size (cm)	Bosniak classification	Diseases treated in the same surgery	Surgical time (min)	Blood loss (ml)	Hospital stay following surgery (day)	Cyst size at 3 months after the surgery (cm)
1	Male	41	6.0	I	–	30	3	2	0
2	Male	48	6.3	I	simple renal cyst	41	5	3	0.5
3	Male	68	7.3	I	–	29	4	2	0
4	Male	55	7.2	I	Renal cell carcinoma	105	40	7	0
5	Male	55	10.5	II	–	25	3	2	1
6	Male	64	6.5	I	Simple renal cysts	45	6	3	0
7	Male	62	5.2	I	–	33	6	2	1
8	Male	52	7.3	I	–	35	7	2	0
9	Male	74	6.5	II	–	42	8	3	1
10	Female	62	7.2	I	–	40	8	2	0
11	Female	72	7.0	I	Simple renal cysts	47	7	3	1.5
12	Female	66	5.8	I	–	29	5	2	0
13	Female	68	5.0	I	–	35	5	2	0.5
14	Female	70	4.8	I	Simple renal cysts	46	10	3	1
15	Female	54	6.3	II	–	38	10	2	0
16	Female	48	7.4	I	–	32	6	2	0

### Laparoscopic renal cyst decortication using methylene blue

**Figure 1:**
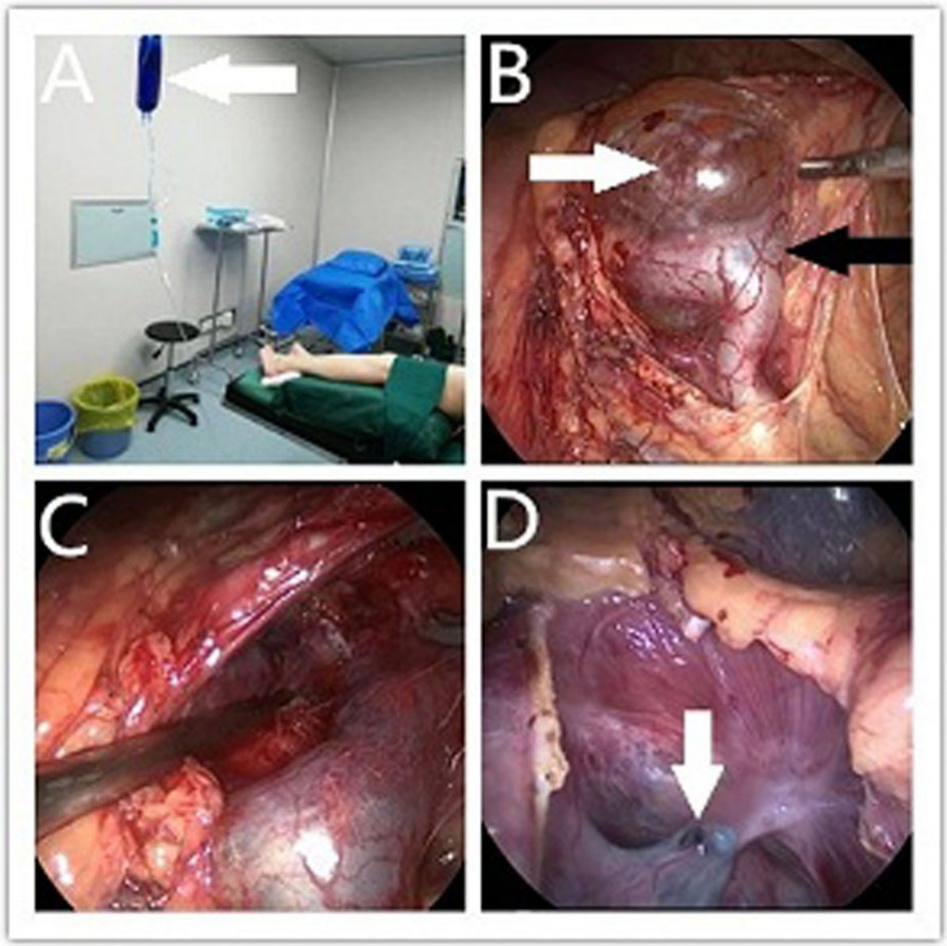
The surgical procedure. (**A**) The ureteral catheter was connected to a mixed solution of 0.2% methylene blue saline (white arrow). (**B**) The cyst (white arrow) was positioned near the collecting system (black arrow). The perfused methylene blue stained the collection system, differentiating it from the cyst wall. (**C**) The renal parapelvic cyst was resected with no secondary damage. (**D**) The cyst was connected to the collection system and leakage of a blue liquid was seen from the cyst wall (white arrow).

All of patients underwent general anesthesia with endotracheal intubation in a lithotomy position, and an F6 zebra ureteral catheter (Shangyi Kangge, Shanghai, China) was inserted retrogradely into the renal pelvis of the affected side. The indwelling catheter and the zebra ureteral catheter were fixed to prevent prolapse. The zebra ureteral catheter was then connected to a mixed solution of 0.2% methylene blue and saline (1000 ml saline plus 2 ml methylene blue) (Fig. 1A). The patient who had a coexisting renal cell carcinoma was then placed in a 45 degree healthy lateral recumbent position prior to undergoing renal parapelvic cyst decortication in addition to a partial nephrectomy through an abdominal approach. The other 15 patients were placed in a lateral position with the side to be operated upward. Retroperitoneal laparoscopic decortication of the renal parapelvic cyst was performed using the procedures of a conventional 3-hole method. The patients who had simple renal cysts received treatment for those cysts during the same surgery. The 0.2% methylene blue solution was infused continuously into the pelvis through the zebra ureteral catheter during the surgery in order to stain the collecting system (Fig. 1B,C). During the procedure, the patients were visually monitored for the presence of methylene blue liquid effluent, which would indicate that the renal collection system was injured.

### Duration and outcome of follow-up

The patients were followed-up every 3 months. During each follow-up visit investigations, such as renal ultrasound scan or enhanced computer tomograph scans were performed. to evaluate mainly the size of residual lesions after surgery. The presence or absence of infection, urinary fistula, or hydronephrosis were also assessed. Those that had coexisting renal stones, were evaluated to ascertain whether the stones had resolves or had recurred.

## Results

All 16 patients in this study successfully underwent laparoscopic surgery and there was no need to change to open surgery. The mean operation time was 40.8 min (range, 25–105 min) and the mean amount of blood loss was 8.3 ml (range, 3–40 ml). In one case, blue colored fluid leaked from the medial wall of the cyst after the lateral wall of the cyst was resected (Fig. 1D). It was determined that this cyst was connected to the collection system that was injured. A running suture using absorbable 4-0 monocryl was immediately used to repair the renal collection system near the wall of the parapelvic cyst until the leakage of blue colored liquid was no longer observed. The cavity of the cyst was then filled with fat from the pedicle of the kidney in order to prevent recurrence^6^. In this patient, the zebra ureteral catheter was removed after 2 weeks, while the other 15 patients had their catheters removed immediately after surgery. A retroperitoneal drainage tube that was inserted to drain any retroperitoneal fluid collection, was removed after confirming that there was no fluid drainage 1–3 days after surgery. The wounds healed very well, and the mean length of post-operative hospital stay was 2.6 days (range, 2–7 days). The wall of the cyst in all 16 cases were biopsied and pathological examination revealed that they were benign lesions. The pathological examination of the patient with a coexisting ipsilateral renal tumor revealed that it was a renal clear cell carcinoma.

**Figure 2:**
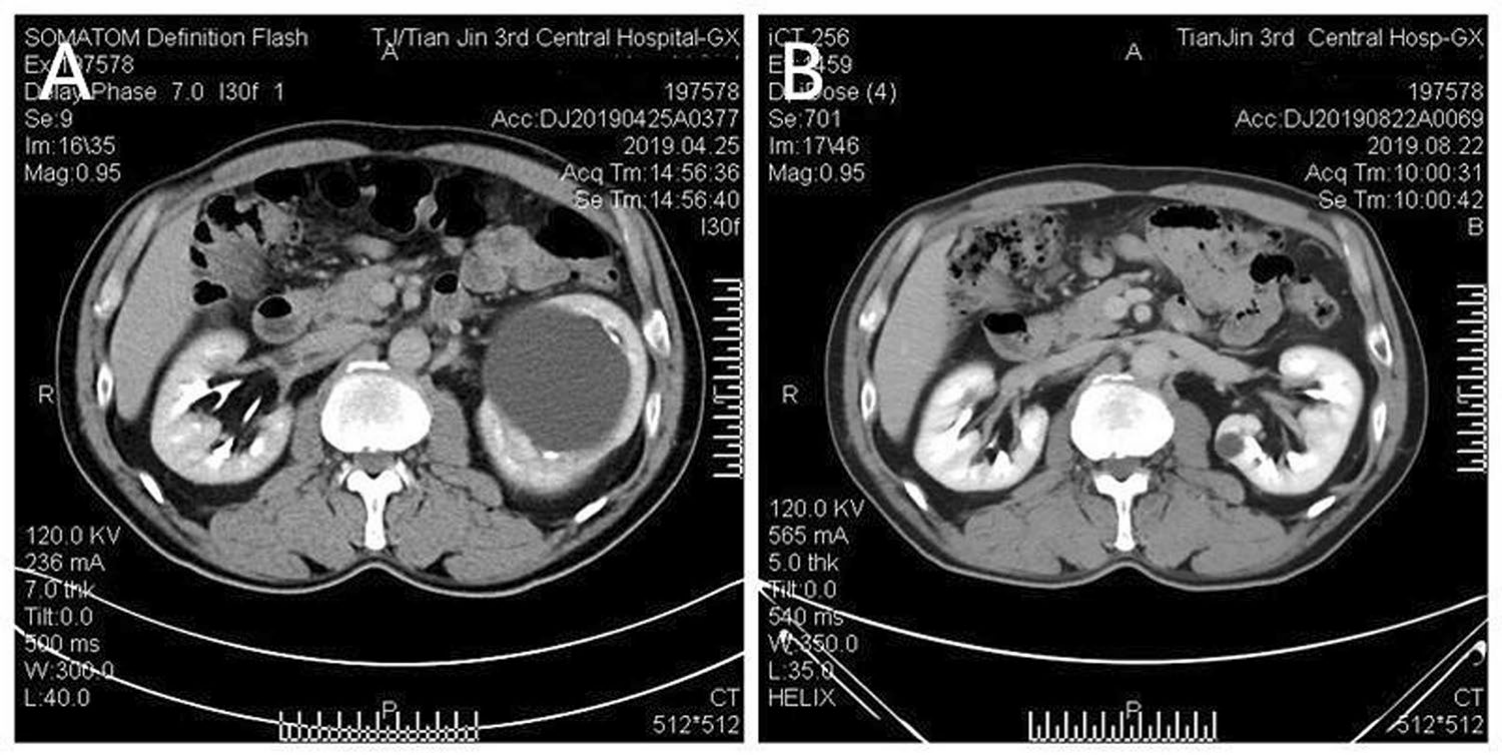
Preoperative and postoperative computed tomography. (**A**) Preoperative computed tomography image of the left renal parapelvic cyst. (**B**) Postoperative computed tomography, 3 months after surgery, showed the cyst had disappeared.

All patients underwent following-up for 3–24 months. The cysts disappeared completely in 9 patients (Fig. 2). However, in the other 7 cases the size of the cysts decreased by more than 50%. Hydronephrosis was significantly alleviated in affected patients. The patient with a 0.6 cm kidney stone passed the stone into the ureter 20 days after surgery, probably because the obstruction was relieved, and the stone was discharged after extracorporeal shock wave lithotripsy. There were no complications related to the administration of methylene blue.

## Discussion

Renal parapelvic cysts occur in the renal sinus and are histologically divided into urinary and non-urinary cysts^7^. It is difficult to distinguish renal parapelvic cysts from hydronephrosis using ultrasonography or ordinary computer tomography; therefore renal parapelvic cyst can be misdiagnosed. Correct diagnosis can be made with enhanced computed tomography. Renal parapelvic cysts are located close to the collecting system and this often causes discomforting features, such as flank pain, hematuria, infection, urinary obstruction, and renin-mediated hypertension^8^. Therefore, surgical intervention is performed more often for renal parapelvic cysts than for simple renal cysts. Secondary kidney stones also occur due to obstruction and infection^9^.

Nephrectomy was previously used as a treatment option for renal cysts^10^. Currently, the methods for the treatment of renal parapelvic cysts include open surgery, percutaneous renal cyst puncture, flexible ureteroscopy, and laparoscopic decortications^11^.

Open surgery is rarely used because it is quite invasive and is associated with more tissue trauma. Percutaneous renal cyst puncture produces less trauma but has a higher recurrence rate^12^. Injecting a sclerosant into the cyst can reduce the recurrence rate. The puncture procedure is difficult because renal parapelvic cyst are located close to the collecting system and the procedure may result in injury to the collecting system and renal blood vessels. Additionally, leakage of the sclerosant can cause severe perinephritis, ureteropelvic junction obstruction, abscess, fever, and other complications^13^.

With the continuous development and improvement of ureteroscopy, incision and drainage of renal cysts using flexible ureteroscopy and holmium laser is becoming increasingly popular. It is associated with minimal trauma and a short postoperative hospital stay^14^. Unfortunately, accurately locating of the cyst is sometimes difficult, especially in patients with thick-walled cysts. Zhixian Wang et al., reported that injecting methylene blue into the cyst during percutaneous renal puncture greatly improved the accuracy of finding cysts and shortened the operation time^15^. However, this type of surgery requires the use of an indwelling double J stent before and after surgery, which brings additional inconvenience to the patient and increases the risk of infection after surgery^16^. Furthermore, this procedure cannot be used in patients with a ureteral stricture and cannot be combined with the simultaneous treatment of simple renal cysts or other kidney lesions.

Laparoscopic renal cyst decortication is safe and effective, especially for large simple renal cysts, and is the gold standard for the treatment of renal cysts^16^. Laparoscopic decortication can also be used in the treatment of renal parapelvic cysts^17,18^, but it requires specialized surgical skills^19^. Renal parapelvic cysts are usually located close to the renal collecting system and the renal blood vessels; therefore, the separation process is associated with the risk of secondary damage, including the probability of vascular injury (1.7%), of visceral injury (0.25%), and renal pelvic injury (0.12%)^20^. It can be difficult to determine if the collecting system is injured during surgery; however, damage to the collecting system can lead to persistent urinary fistula after surgery. According to our results, we achieved good outcomes with this method, some advantages of out method are: (1) After injecting methylene blue through the ureteral catheter, the collection system will stain blue which improves the visibility of the collecting system relative to the cyst wall, and allows for complete resection of the cyst wall. The ureteral catheter plays a guiding and a protective role in the ureter; therefore, we can greatly avoid secondary ureteral damage during surgery. (2) If the renal cyst is connected to the collecting system, identifying the renal cyst during traditional laparoscopic renal cyst decortication surgery can be difficult. If continuous exudation of urine is seen in the drainage tube after surgery, additional surgery will be required to repair the collecting system. This would cause the patient more pain. However, if this situation is encountered while using our methodology, leakage of a blue colored liquid from the wall of the cyst can be seen during surgery, and we can simultaneously repair the injured wall at the same time. The zebra ureteral catheter inserted during the operation can remain in situ for 2 weeks and acts as an internal drain. The ureteral catheter can be removed directly without the aid of a cystoscope. (3) If other lesions are present, they can be treated along with this procedure during the surgery. In this study, one patient with renal cell carcinoma and four patients with simple renal cysts also underwent relevant treatment for their respective conditions during the same surgery. (4) Compared with flexible ureteroscopy, there is no need to use a double J stent before and after surgery, and this reduces the cost of treatment^21^. Patient with ureteral stricture can also undergo this surgical approach. Francesco et al., reported that the infection rate following flexible ureteroscopy was about 7.7%^22^, but our surgical procedure had a lower infection rate, as no patient in this study had infections after surgery. (5) Methylene blue is a tricyclic phenothiazine that inhibits guanylate cyclase. It has been used in several fields of medicine. The most common side effects of methylene blue administration are self-limiting blue-green cutaneous discoloration and urine discoloration^23^. In our study, methylene blue entered the renal pelvis or calyces and flowed out through the ureter, without entering the general circulation. Even if a small amount had entered the body, methylene blue is a mature drug that can be excreted in the urine with little metabolism or side effects^24^, and no special treatment would be required. Compared with enhanced computer tomography, methylene blue is not radioactive and can be used continuously during surgery.

## Conclusions

Renal parapelvic cysts are often encountered by urologists and they are difficult to manage. Laparoscopic renal cyst decortication is still the preferred treatment; however, it is associated with the risk of secondary injury to nearby structures^20^. In this study, the combination of methylene blue and laparoscopy achieved good results and was found to be a safe, effective, and practical method for the treatment of renal parapelvic cysts. However, further studies with longer follow-up periods are required to verify the effectiveness of this method.

## Data Availability

The datasets used and/or analyzed in this study are available from the corresponding author upon reasonable request.
